# Supplementary material for: Safety of FOLFIRI + Durvalumab +/− Tremelimumab in Second Line of Patients with Advanced Gastric Cancer: A Safety Run-In from the Randomized Phase II Study DURIGAST PRODIGE 59
Source: Biomedicines. 2022 May 23;10(5):1211. doi: 10.3390/biomedicines10051211 (PMC9138589; doi:10.3390/biomedicines10051211)
Supplement: Supplementary file 1 [file biomedicines-10-01211-s001.zip › Supplementary Data S2.pdf]

Supplementary Data S2. Treatment-not related adverse events.

n, %	Arm A (Folfiri + Durvalumab) (n = 8)		Arm B (Folfiri + Durvalumab + Tremelimumab) (n = 3)	
	**Grade 1–2**	**Grade 3–4–5**	**Grade 1–2**	**Grade 3–4–5**
**Patients exhibiting at least one adverse event**	**7 (87.5%)**	**4 (50.0%)**	**2 (66.7%)**	**1 (33.3%)**
**Ear and labyrinth disorder**	**1(12.5%)**	-	**1 (33.3%)**	-
Vertigo	1(12.5%)	-	1 (33.3%)	-
**Skin and subcutaneous tissue disorders**	**1 (12.5%)**	-	-	-
Dry skin	1 (12.5%)	-	-	-
**Nervous system disorders**	**4 (50.0%)**	**1 (12.5%)**		**1 (33.3%)**
Peripheral sensory neuropathy	4 (50.0%)	1 (12.5%)	-	-
Syncope	-	-	-	1 (33.3%)
**Gastrointestinal disorders**	**2 (25.0%)**	**2 (25.0%)**	**3 (100.0%)**	
Constipation	-	-	1 (33.3%)	-
Abdominal pain	1 (12.5%)	-	2 (66.6%)	-
Dysphagia	-	2 (25.0%)	-	-
Dyspepsia	1 (12.5%)	-	-	-
Vomiting	-	1 (12.5%)	-	
**Musculoskeletal conditions**	**1 (12.5%)**	-	**1 (33.3%)**	-
Back pain	1 (12.5%)	-	-	-
Generalized muscle weakness	-	-	1 (33.3%)	-
**Respiratory, thoracic and mediastinal disorders**	**3 (37.5%)**	**2 (25%)**	**1 (33.3%)**	-
Dyspnoea	1 (12.5%)	1 (12.5%)	-	-
Cough	2 (25.0%)	-	1 (33.3%)	-
Aspiration	-	1 (12.5%)	-	-
**Infections and infestations**	**3 (37.5%)**	**2 (25.0%)**	**1 (33.3%)**	**1 (33.3%)**
Abdominal infection	-	-	-	1 (33.3%)
Urinary tract infection	1 (12.5%)	1 (12.5%)	-	-
Lung infection	-	1 (12.5%)	-	-
Prostate infection	1 (12.5%)	-	-	-
Tooth infection	1 (12.5%)	-	1 (33.3%)	-
**Blood and lymphatic system disorders**	**1 (12.5%)**	-	-	-
Lymphopenia	1 (12.5%)	-	-	-
**Investigations**	**2 (25.0%)**	**1 (12.5%)**	-	-
Gamma-glutamyltransferase increased	1 (12.5%)	1 (12.5%)	-	-
Alkaline phosphatase increased	1 (12.5%)			
**Metabolism and nutrition disorders**	**1 (12.5%)**	-	**1 (33.3%)**	-
Anorexia	-	-	1 (33.3%)	-
Hyperglycaemia	1 (12.5%)	-	-	-
**General disorder**	1 (12.5%)	-	-	**1 (33.3%)**
Fatigue	-	-	-	1 (33.3%)
Fever	1 (12.5%)	-	-	-

The total of adverse events could be superior to the total number of patients since some patients could have more than one adverse event.
